# Nurse Researchers’ Experiences and Perceptions of Generative AI: Qualitative Semistructured Interview Study

**DOI:** 10.2196/65523

**Published:** 2025-08-25

**Authors:** Ruifu Kang, Zehui Xuan, Ling Tong, Yanling Wang, Shuai Jin, Qian Xiao

**Affiliations:** 1 School of Nursing, Capital Medical University, No.10 Xi-tou-tiao, You-an-men Wai, Feng-tai District, Beijing, 100069, China

**Keywords:** generative artificial intelligence, large language model, nurse researcher, nursing research, qualitative study

## Abstract

**Background:**

With the rapid development and iteration of generative artificial intelligence, the growing popularity of such groundbreaking tools among nurse researchers, represented by ChatGPT (OpenAI), is receiving passionate debate and intrigue. Although there has been qualitative research on generative artificial intelligence in other fields, little is known about the experiences and perceptions of nurse researchers; this study seeks to report on the topic.

**Objective:**

This study aimed to describe the experiences and perceptions of generative artificial intelligence among Chinese nurse researchers, as well as provide a reference for the application of generative artificial intelligence in nursing research in the future.

**Methods:**

Semistructured interviews were used to collect data in this qualitative study. Researchers mainly conducted interviews on the cognition, experience, and future expectations of nurse researchers regarding the use of generative artificial intelligence. Twenty-seven nurse researchers were included in the study. Through purposive sampling and snowball sampling, there were 7 nursing faculty researchers, 10 nursing graduate students, and 10 clinical nurse researchers. Data were analyzed using inductive content analysis.

**Results:**

Five themes and 12 subthemes were categorized from 27 original interview documents as follows: (1) diverse reflections on human-machine symbiosis, which includes the interplay between substitution and assistance, researchers shaping the potential of generative artificial intelligence, and acceptance of generative artificial intelligence with alacrity; (2) multiple factors of the usage experience, including individual characteristics and various usage scenarios; (3) research paradigm reshaping in the infancy stage, which involves full-process groundbreaking assistive tools and emergence of new research paths; (4) application risks of generative artificial intelligence, including intrinsic limitations of generative artificial intelligence and academic integrity and medical ethics; and (5) the co-improvement of technology and literacy, which concerns reinforcement needs for generative artificial intelligence literacy, development of nursing research generative artificial intelligence and urgent need for artificial intelligence–generated content detection tools. In this context, the first 4 themes form the rocket of the human-machine symbiosis journey. Only when humans fully leverage the advantages of machines (generative artificial intelligence) and overcome their shortcomings can this human-machine symbiosis journey reach the correct future direction (fifth theme).

**Conclusions:**

This study explored the experiences and perceptions of nurse researchers interacting with generative artificial intelligence, which was a “symbiotic journey” full of twists and turns, and provides a reference and basis for achieving harmonious coexistence between nurse researchers and generative artificial intelligence in the future. Nurse researchers, policy makers, and application developers can use the conclusions of this study to further promote the application of generative artificial intelligence in nursing research, policy making, and product development.

## Introduction

### Generative Artificial Intelligence Holds Great Potential in Nursing Research as a Tool

Nursing research, an integral part of the nursing profession, enhances the knowledge base of nursing practice, refines the methodologies used, supports the health of the whole human lifecycle, develops health strategies for specific populations, and establishes needs-focused health care systems [1]. At each stage of nursing research, appropriate research tools are essential. Over time, information technologies have increasingly penetrated the field of nursing research, with mature tools such as databases and statistical software having a profound influence on nursing scientific research [2]. Representing a new technological era, artificial intelligence (AI) has been repeatedly mentioned in recent scientific advances. For instance, AI tools have emerged in evidence-based research for semiautomated literature screening and data extraction [3]. These tools allow researchers to extricate themselves from the tedious and monotonous aspects of research and instead focus on more innovative elements, thereby enhancing the efficiency of evidence-based studies. Generative artificial intelligence (GenAI) [4], as a cutting-edge form of AI, has attracted widespread attention and discussion among scientific researchers worldwide following the release of ChatGPT (OpenAI) on November 30, 2022.

### GenAI Introduction

GenAI refers to an AI capable of generating various data types, such as text, images, videos, and audio. GenAI learns from large amounts of data to capture intrinsic patterns to generate similar new data [5]. Currently, there is much discussion about text-to-text GenAI and text-to-image GenAI in scientific research. Text-to-text GenAI is the earliest and best known. It can respond to or generate textual content according to human requests. Based on large language models, its representative products include the aforementioned ChatGPT, ERNIE Bot (Baidu), Gemini (Google DeepMind), and DeepSeek (DeepSeek). Another is text-to-image GenAI, which can produce images that match the user’s textual descriptions; notable examples include Stable Diffusion (Stability AI), Midjourney (Midjourney), and DALL-E (OpenAI). In addition, there is GPT-4o, a powerful multimodal GenAI capable of processing various types of information, such as text and images individually [6]. These products also include reasoning models such as DeepSeek-R1 and GPT-o1 that have recently received extensive attention. These GenAI generate text and images based on text and are capable of some degree of expansion and “creativity” on the text provided by users. The “creativity” of GenAI, which distinguishes them from other AI, suggests that they have the potential to become groundbreaking new research tools in scientific work [7]. Researchers have extensively explored and discussed the application of GenAI in scientific research, focusing mainly on high-performance large language models and text-to-image GenAI.

### Advantages of GenAI

One of the key advantages of GenAI is its ability to learn from massive amounts of data, making it highly scalable and adaptable to different situations. It can generate easy-to-understand natural language and exquisite images in real time through simple text prompts. In addition, GenAI can also be fine-tuned for specific domains, making it more knowledgeable in certain areas [8]. For example, ChatGPT can pass the nursing licensure exam in Chinese Taiwan [9]. Moreover, ChatGPT can answer complex questions, provide reliable information about diseases, and answer medical queries. It has the potential to complement the expertise of health care providers to improve clinical decision-making and patient care [10]. In the field of research, ChatGPT has proven to have the ability to generate scientific papers that resemble authentic papers written by human researchers [11]. The literature review generated by Google Bard seems promising overall [12]. There is research that used Midjourney to generate scientific illustrations on tornado dynamics and found that text-to-image GenAI has great potential for creating scientific illustrations despite its limitations [13]. These all demonstrate the powerful capabilities of GenAI in academic research.

### Limitations of GenAI

At the same time, GenAI also has some shortcomings. On one hand, GenAI includes the inability to access real-time information and issues with the accuracy of information. The information contained within the model itself still depends on the cutoff time of training. Therefore, its inability to access new information or the internet in real-time makes the timeliness of the information a concern [14]. The accuracy issue is manifested in the case of fabricating answers that are “AI hallucinations,” a phenomenon that exists with GPT-4 [15]. On the other hand, as GenAI is used in nursing research, nurse researchers are beginning to worry that using GenAI (such as ChatGPT) as an author promotes academic dishonesty and allows students to generate research papers without doing their research by themselves [16]. Although ChatGPT has been published as a co-author in a peer-reviewed academic paper [17]. Most journals all agreed that ChatGPT cannot be listed as an author in academic papers, arguing that including it as an author contradicts academic ethics [18-20]. Some researchers have argued that the practice of writing using GenAI undermines ethical integrity in nursing and that reliance on ChatGPT in nursing education may produce nurses who do not adhere to ethical values, lack a sense of trustworthiness, and are overconfident in handling complex cases [21].

### GenAI’s Flaws Do Not Overshadow Its Strengths

Despite these limitations, GenAI’s ability to analyze large amounts of data was several times faster than humans, so it could be used for cursory searches to quickly familiarize itself with the literature, thus helping researchers save more time [22]. Furthermore, ChatGPT can help find scientific references, write the methods section of a research paper, and suggest appropriate statistical analyses, among other things [23]. Copilot (Bing AI; Microsoft) can automate data extraction for systematic reviews, providing additional verification for novice researchers [24]. Nature reported that scientists used ChatGPT as a research assistant to help them organize their thoughts, get feedback, write code, and summarize research literature [25]. Scientific researchers’ widespread use of GenAI in their daily work is evident. Using GenAI rationally can enhance scientific research efficiency [26].

### The Objectives and Significance of the Research

Hence, the judicious use of GenAI will help nurse researchers be more efficient in their research work and benefit them by facilitating learning, improving digital literacy, and encouraging critical thinking about the integration of GenAI for nurse researchers. As the medical staff who have the most frequent contact with patients, nursing research generally focuses on direct interaction with patients and emphasizes humanistic care. All these determine the differences between nursing research and other medical research. Therefore, it is necessary to explore nurse researchers’ subjective experiences and perceptions of GenAI to promote the rational use of GenAI in nursing research. However, there is currently no such research available. This study aimed to describe nurse researchers’ experiences and perceptions of the use of GenAI in nursing research as well as to explore the distress and concerns about using GenAI via qualitative research methods to provide a reference for the ethical use and development of GenAI in nursing research.

## Methods

### Design

This study used a qualitative descriptive approach to obtain detailed descriptions of participants’ experiences via semistructured interviews. Interviews were conducted in 2 forms: face-to-face and video calls. The reason for choosing this approach was to enrich the phenomena and obtain credible data that closely reflect the true inner thoughts of nurse researchers, given how little we know about the experiences and perceptions of applying GenAI to nursing research. The study reporting followed the Consolidated Criteria for Reporting Qualitative Research Checklist (COREQ) [27] (Multimedia Appendix 1).

### Setting and Participants

Purposive and snowball sampling were used to recruit nurse researchers from 2 hospitals and 4 nursing institutions in China for 5 months, from August 2023 to December 2023. In this qualitative study, purposive sampling was first used to select participants who met specific criteria relevant to the research topic, ensuring the sample’s relevance and depth. Snowball sampling was then applied to reach hidden or hard-to-access groups, enriching the data diversity. This combination aimed to achieve information saturation, which aligns with the nature of qualitative research [28]. First, the first author contacted potential participants via WeChat (Tencent) to briefly introduce the purposes and methods of this study and to understand their experiences using GenAI. If they met the eligibility criteria, their consent was sought along with scheduling the interview time. All recruited participants volunteered to participate in our study and could communicate in Mandarin. No potential research candidates refused to participate. The authors have experience learning or practicing in the participants’ workplaces, so the interviews were based on mutual trust, which improved the trustworthiness of the data [29].

### Inclusion and Exclusion Criteria

The study participants included in this research all have experience with GenAI. Inclusion criteria for clinical nurse researchers were (1) over 1 year of nursing research experience, (2) at least a bachelor’s degree, and (3) at least a midlevel professional title. The criteria for nursing faculty researchers were (1) over 1 year of nursing research experience, (2) a minimum of a master’s degree, and (3) at least a midlevel professional title. Nursing graduate students were included if they (1) had over 1 year of nursing research experience and (2) were currently enrolled in an MSc or PhD program in nursing. The exclusion criteria were (1) only had experience with nongenerative AI and (2) had a mental disorder that prevented them from answering questions properly.

### Data Collection

For participants in Beijing, data were collected through face-to-face interviews with 2 researchers, postgraduate students who have taken qualitative research courses and have experience in interviewing. They are a man and a woman engaged in nursing informatics research, and they are professionally trained in active listening and nondirective questioning. For those outside Beijing or physically constrained, interviews were conducted through Tencent Meeting (Tencent). Based on the discussion of the research team and consultation with qualitative experts, the preliminary interview questions were determined. After that, we conducted preinterviews with 2 student nurse researchers to rule out possible problems. After these steps, the final interview outline was determined, including (1) what have you done with GenAI in nursing research? (2) What do you feel about using GenAI in nursing research? (3) What factors influence nurse researchers to use GenAI and what are the difficulties and barriers? (4) What do you think about GenAI-assisted writing or co-authoring articles with GenAI for publication? (5) What are your requirements for GenAI and what is your perspective on its future development? The interviews were conducted in a quiet and undisturbed environment to ensure the continuity of thought for both the authors and the participants. Before the interviews, the researchers reiterated the research purpose and interview contents to the participants, who signed the informed consent on the spot. During the interviews, the authors used responsive, interpretative, summarizing, and repeating methods to encourage the participants to share their experiences and needs in using GenAI in nursing research, thereby minimizing the impact of the authors’ personal views on participants’ thinking. The content of the interviews was recorded anonymously using the recording function of Tencent Meeting or a voice recorder [30]. The 2 authors had different responsibilities during the interviews, with 2 co–first authors communicating and transcribing the interviews. Data collection was stopped when no new themes were generated [31]. Then, one additional interview was continued until sampling was stopped after no new themes emerged from the analysis of information from the additional interviews to prove that the data had reached saturation.

### Data Analysis

In this study, an inductive content analysis approach [32] was used to scrutinize the data and attain an in-depth comprehension of the experiences and perceptions of nurse researchers in GenAI. Initially, the first author used the speech-to-text platform to automatically transcribe the raw data, which was then double-checked by 2 authors to generate 27 Word documents. These Word documents were imported into NVivo 14 software (Lumivero) for further analysis. The authors read the documents repeatedly in detail to understand and get a holistic sense of the interview data. In this process, they naturally formulated a preliminary inductive framework based on the research objectives. The authors meticulously searched the data for answers to the research questions, selecting a single concept or sentence as a unit of meaning. After identifying the original expressions, the authors simplified and coded them, grouping codes with similar meanings and assigning them descriptive labels; any conflicts during this process were discussed between the 2 authors and they listened to the original recordings. The supervising researcher, another author, evaluated the credibility of the entire coding and generalization process, joining the data analysis at random times to ensure consistency. These categories were subsequently merged to form new ones, constantly ensuring their alignment with the research questions. This process continued until no additional categories could be formed. The researchers conducted separate analyses for each research question, and the incremental findings were disseminated for discussion and proposed modifications at the weekly meetings, with all authors recognizing the final results.

### Rigor and Reflexivity

This study ensures rigor from 4 aspects: credibility, dependability, transferability, and confirmability [33]. This study conducted comprehensive interviews with the research participants in a quiet environment, ensuring the credibility and dependability of the research through follow-up visits with the participants and regular discussions with other researchers in the study group to verify and discuss the analysis results. The transferability of the study was ensured through detailed descriptions of the individual information of the research participants and the research context, as well as by including different groups of nurse researchers, such as nursing postgraduates, faculty from nursing institutions, and clinical nurse researchers from various provinces in China. Finally, we introduced an audit trail in the study to ensure the confirmability of the research results.

The researchers kept detailed records of the research process and related reflections through the memo and log feature in NVivo software and repeatedly reviewed whether their results were influenced by personal characteristics or subjective judgments, engaging in personal reflexivity. On the other hand, in regular group discussions, each member of the research team was able to question and discuss the researchers’ viewpoints, achieving collaborative reflexivity [34].

### Ethical Considerations

The study received ethical approval from the ethics committee of Capital Medical University (Z2022SY027). Informed consent was obtained from all participating pairs. All participants were notified that study involvement was voluntary and they could withdraw at any time without repercussions. In addition, we did not provide any economic compensation to any participants. All original interview data and participants’ information were anonymized and stored on an encrypted cloud drive to maintain confidentiality.

## Results

### Characteristics of Participants

A total of 27 nurse researchers participated in this study, including clinical nurse researchers (n=10), nursing graduate students (n=10), and nursing faculty researchers (n=7), all with varying degrees of research responsibilities, from hospitals and nursing institutions in 4 provinces and a special administrative region (Beijing, Shanghai, Jilin, Henan, and Hong Kong) of China. The quotations were labeled with “C,” “S,” and “F” to represent “clinical nurse researchers,” “nursing graduate students,” and “nursing faculty researchers,” respectively, followed by the number of participants. The total length of the interviews was 1881 minutes, ranging from 24 to 131 minutes, with an average of 70 (SD 18.5) minutes per participant. Detailed demographic characteristics of the nurse researchers are shown in Table 1. The mean ages of the clinical nurse researchers, nursing graduate students, and nursing faculty researchers were 33.70 (SD 5.12), 25.10 (SD 2.85), and 34.71 (SD 7.95), respectively.

**Table 1.** Demographic characteristics of nurse researchers.

Characteristics	Participants (N=27)
Age (years), mean (SD)	30.78 (6.81)
Sex, n (%)	
Male	5 (19)
Female	22 (81)
Identity, n (%)	
Clinical nurse researcher	10 (37)
Nursing graduate student	10 (37)
Nursing faculty researcher	7 (26)
Educational background, n (%)	
Bachelor	1 (4)
Master	10 (37)
PhD	6 (22)
Maste’sr degree candidate	5 (19)
Doctoral candidate	5 (19)
Professional title, n (%)	
Senior nurse	1 (4)
Nurse-in-charge	8 (30)
Deputy nurse director	1 (4)
Associate professor	3 (11)
Lectorate	4 (15)
NA^a^	10 (37)
Department, n (%)	
Neurology department	3 (11)
Rehabilitation department	1 (4)
Nursing department	1 (4)
Intensive care unit	3 (11)
International department	1 (4)
Neurosurgery department	1 (4)
School of nursing	17 (62)

a NA: Because students do not have professional titles, it is “not applicable.”

### Five Major Themes

**Figure 1.**
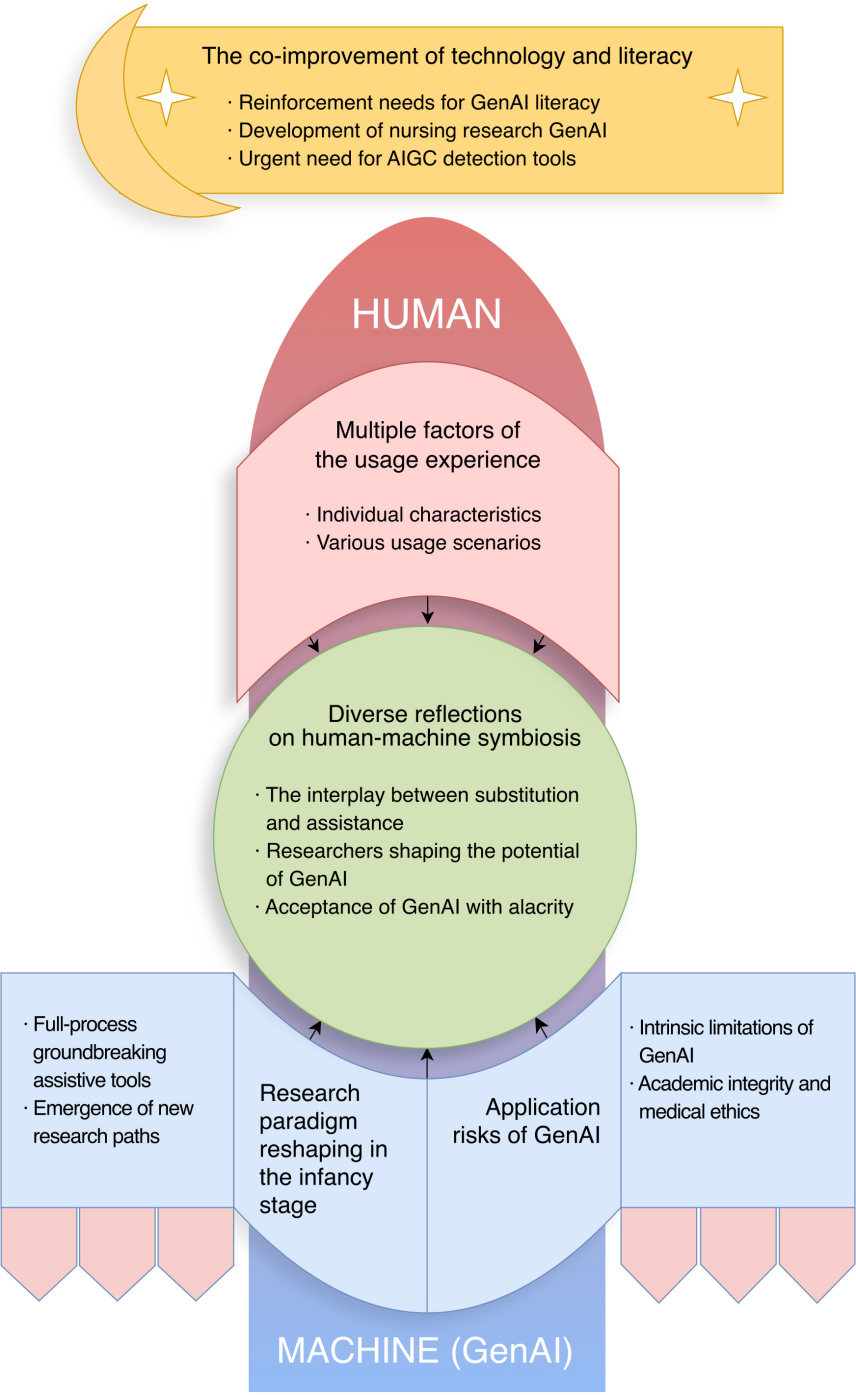
Nurse researchers’ experiences and perceptions of GenAI. AIGC: AI-generated content; GenAI: generative artificial intelligence.

The results revealed 5 key themes in the winding journey of human-machine symbiosis. We call it the “rocket” model. Among these 5 themes, "multiple factors of the usage experience” belongs to the human aspect, while “research paradigm reshaping in the infancy stage” and “application risks of GenAI” pertain to the machine (GenAI) aspect. Humans and machines, through “diverse reflections on human-machine symbiosis,” together form the rocket that propels this journey. In this journey, humans steer the direction from the rocket’s head, while GenAI serves as the engine driving the rocket. Only by humans fully leveraging the advantages of GenAI and overcoming its shortcomings can this journey of human-machine symbiosis ultimately head in the right direction (“The co-improvement of technology and literacy”; Figure 1).

### Theme 1: Diverse Reflections on Human-Machine Symbiosis

This theme encompasses the participants’ deep reflections triggered by the application of GenAI to nursing research. On the other hand, this theme also aims to appeal to nurse researchers to make the best use of its convenience while maintaining a critical vision and self-active thinking, and treating the run-in between humans and machines with a peaceful mind.

#### The Interplay Between Substitution and Assistance

This subtheme highlights the different perspectives of nurse researchers on the work that was replaced or supported by GenAI. GenAI performed mechanical tasks well and completed many intellectual tasks, such as innovation and planning, which the participants recognized. Some participants feared being replaced by GenAI in the future because they felt insignificant in the face of its intelligence.

*I have a sense of crisis. Because I have seen a lot published in the Lancet... if one-day GenAI surpasses my ideas and it quickly completes the work I have been doing for a long time, which is a shock to me, and I would feel anxious.*

[C7]

Conversely, another group of participants was optimistic, believing that the power of GenAI could inspire researchers to work tirelessly to continuously improve themselves, resulting in a positive relationship between reciprocal promotion and achievement.

*I hope that one day the content generated by GenAI can be an inspiration or source for me to continue to learn and improve my competitiveness. I think so.*

[S10]

#### Researchers Shaping the Potential of GenAI

This subtheme implies that the accumulation of knowledge and experience by nurse researchers and the performance of GenAI usually go hand-in-hand. More than half of the participants reported that users with a solid scientific foundation and strong logical thinking are more likely to inspire better academic performance in GenAI. Conversely, if the user has inadequate skills or lacks self-discipline, this can play a negative role. Therefore, GenAI is a double-edged sword, depending on how nurse researchers use it.

*If you already can think spontaneously and have some basic skills in reading literature, such as understanding logical structure, writing style, persuasiveness of the data, and so on, GenAI will be a valuable tool.*

[F2]

*I think GenAI is like a buff (an extra toolkit of gainful effects) in games, but it is hard to rely on it to achieve your goals if you are not skilled enough. ... It is like a sword, and how much effect you can get out of it depends on your skill.*

[F6]

#### Acceptance of GenAI With Alacrity

Participants recalled their first experience with GenAI, either being recommended to experience it or actively exploring it after noticing media coverage, which demonstrated a strong exploratory desire to explore and an openness of nurse researchers to new things. Next, they shared their feelings and attitudes at the time and were pleasantly surprised by the capabilities of GenAI, stating that it was worth promoting and becoming an integral part of the work, which expressed their high affirmation.

*I learned about GenAI at a school reunion where someone told me about the tool. That is when GenAI was in a blaze of publicity, so I started using it. ... I think we should accept it with an open and cooperative mind because it was a creature of The Time; we should keep up with the development of the information era.… Yes, I see it as part of my work and life, and I feel good about it... I think that most people taste the benefits of GenAI and then indulge in it.*

[C8]

### Theme 2: Multiple Factors of the Usage Experience

The working environment, educational background, and research experience of the research participants were different. While the differences in individual characteristics and usage scenarios ultimately led to different experiences of nurse researchers with GenAI.

#### Individual Characteristics

Nurse researchers in our study included clinical nurse researchers, nursing graduate students, and nursing faculty researchers. They differ in age, gender, background, and educational experience, resulting in different perceptions and experiences of GenAI. The minority of participants reported that the inherent female predominance of the nursing profession was a likely reason for this group being less sensitive to GenAI information, possibly due to perceived differences in digital literacy, with women believed to have lower levels than their male counterparts.

*It is a fact that boys are more interested in technology. I know a male teacher in our college who uses ChatGPT to write articles for the official account. ... It is not that all girls do not like technology, but the sensitive part by nature due to gender is really different.*

[F2]

Some nursing faculty were aware of the prevalence of GenAI use among graduate students, who used it to sort literature, modify abstracts, and polish English papers, which may be attributed to young people’s heightened inclination to explore and learn new things.

*When I was the headteacher of first-year students, I knew that many undergraduate and graduate students were actively learning these technologies (using GenAI), and their learning ability is surprisingly good. For example, they used it to sort literature, polish papers and even consider it as Baidu (the world’s largest Chinese search engine).*

[F4]

#### Various Usage Scenarios

In this subtheme, participants objectively described their experiences of research assignments using GenAI, such as analyzing statistical data, providing the latest developments, and asking about the worldly wisdom of the research process. In these scenarios, the research participants have vastly different and rich experiences. In addition, the usage cost of their application scenarios also determines whether they will use GenAI.

*I asked GenAI about research developments in the field, such as new terminologies and new statistical methods, when I read the literature, and then it helped me organize the relevant knowledge in the field.*

[S3]

*The brain can determine whether the cost of habit change is proportional to the benefit; if someone learns quickly, then the input cost is low, but I have not transferred this habit (using GenAI).… If once I find it works, even though I am not comfortable sitting on this seesaw, but if one day the benefits are big enough, I might be able to swing over, you know?*

[F6]

### Theme 3: Research Paradigm Reshaping in the Infancy Stage

Taken as a whole, the majority of participants gave an objective evaluation of the effects and contributions of GenAI applied to nursing research. They believed that GenAI had progressively become the right hand of nurse researchers, undeniably simplifying the scientific research process. It can be argued that the research process of nurse researchers was unconsciously influenced by GenAI, although in the infancy stage, it was also conceivable to reshape the paradigm of nursing research in the long run.

#### Full-Process Groundbreaking Assistive Tools

This subtheme considers GenAI-boosting nursing research as the core concept and explores its capacity for satisfactory academic support. Different participants believed that GenAI can play a role in different aspects of nursing research, such as writing statistical code, polishing the language of papers, and quickly collecting literature.

*After all, if we with a medical and nursing background, don’t have a foundation in computer science or code, it will take a relatively long time to get started with R language and Python. The advantage of using GenAI is that you can actually ask it to do things in a commanding tone, and it will tell you the code, which is very efficient.*

[F4]

*Regarding the issue of language polishing, it largely saves you time. If I ask a company to do the polishing, it may cost a lot of money. But now, I may only need three or four minutes to do the polishing, thus saving both time and money.*

[C8]

*GenAI can collect information from literature and summarize it, and then present this information in a scientific and academic paper manner. This is of great convenience to us.*

[S7]

In addition, most participants reported its benefits as an innovative tool to improve research methods, efficiency, quality, and most importantly, to provide researchers with refreshing ways to inspire and expand their ideas.

*It addresses the knowledge gap at home and abroad. For example, I recently had to review some math-oriented articles, but I don’t understand these formulas, which looks daunting, so I have to rely on GenAI.*

[F3]

*I think it’s wonderful that it thinks more holistically than we do. For example, sometimes we are limited by fixed thinking, which reminds us to think from a different perspective, and you may be inspired.*

[F5]

#### Emergence of New Research Paths

Participants felt that GenAI had molded new paths for nursing research, which could be understood as an incubation process for innovative research directions. It was greatly beneficial to nurse researchers in offering unexpected inspiration and broadening study ideas, thus demonstrating an appealing ability to break down knowledge barriers. Even some participants expressed their high praise for GenAI, claiming that its emergence was epoch-making and, to some extent, an alternative to traditional nursing research paradigms.

*I think it is good and still necessary in the development of artificial intelligence, a landmark product, which is the progress of human civilization. Especially in English communication, generative features may be better.*

[C4]

*You can really feel this development, it was very shocking at the very start. Because you find that people need to spend a lot of time and energy to do some things, for it may be a few seconds, or even a second.*

[F2]

### Theme 4: Application Risks of GenAI

As we all know, GenAI has many shortcomings, such as AI hallucinations, poor timeliness, and payment required. These limitations restrict the application scenarios of GenAI to a certain extent. At the same time, the application of GenAI in nursing research will also bring academic integrity and medical ethics issues.

#### Intrinsic Limitations of GenAI

For another, the student-dominated participants felt that the advanced version of GenAI, although very attractive, was expensive and not cost-effective for current usage needs. Thus, the high cost was another barrier, especially for low-income students.

*The fourth generation of this model (GPT-4) has to be paid for and is quite expensive. It is not necessary to pay for my current needs; at least, I do not rely on it very much at the moment.*

[S5]

Furthermore, participants recognized that GenAI would produce misleading information, requiring them to spend more time verifying the authenticity of responses, leading to a loss of trust and even irritation. They also identified shortcomings in data security, cross-cultural debugging, political hurdles, and timeliness of responses.

*I think the primary drawback of GenAI is inaccurate content. Even when looking for objective information, sometimes what GenAI says seems reasonable, but I am not sure, I have to check the literature myself.*

[S7]

Moreover, participants raised the issue of feeling out of control over the content generated by GenAI due to its hidden operating logic, thus questioning its interpretability.

*When the database was built, we were asked not to use unverified data, and even if we reluctantly accepted, we could only see the final result, but did not know the logic behind it... We still need to see a professional judge.*

[C3]

Beyond the issues of information accuracy and interpretability, nursing, as a discipline closely related to humanistic care, has led nurse researchers to express concerns that GenAI itself cannot understand emotions.

*GenAI may not be able to answer questions related to emotions. It may only deal with things based on experience and lack emotional depth.*

[S1]

#### Academic Integrity and Medical Ethics

Most participants commented that the potential for scientific misconduct and medical ethical dilemmas was the primary scruple in using GenAI. Sticking to the bottom line of science and academic integrity was a prerequisite for their commitment to collaborate with novel tools or technologies to support scientific research. As awareness of GenAI in nursing research gradually increased, participants were concerned that it might disrupt the research environment and allow poor-quality academic work to be disseminated by the convenience of GenAI. Meanwhile, deeper issues arose regarding whether GenAI could be author-compliant and the attribution of responsibility for GenAI-authored works. Most participants agreed that the decision was made depending on the actual contribution of GenAI and the target journal’s detailed requirements.

*My concern is that if someone does not have their heart in the right place, they might take advantage of the convenience of GenAI to step on the red line, so for the sake of the overall ecology of the academic circle, I tend not to use it.*

[S10]

There are also some nurse researchers who are worried about the ethical risks of GenAI and thus dare not use GenAI for scientific research work.

*Since the work of GenAI cannot be completely transparent at present, if I use it for some research work, even if I declare the scope of use, I may still be questioned about the proportion of my work in the final results. Therefore, I will not use GenAI for scientific research work.*

[S1]

Some nurse researchers believe that the use of GenAI will create unfairness between nurse researchers who use GenAI and those who do not.

*The possibility of someone using it means that someone is not using GenAI. This is not a common situation, and it gives the impression that not everyone is being treated equally in this matter.*

[S8]

With these concerns in mind, some participants argued that researchers should capitalize on the positive aspects of GenAI and not be banned for its negative.

*I know that most universities discourage its use. Otherwise, they would not develop anti-AI plugins because writing papers in GenAI is inherently opportunistic. ... But we should see the positive side of it; after all, GenAI is a convenient tool, so why not use it?*

[F2]

### Theme 5: The Co-Improvement of AI Literacy and Technology

The final theme to emerge from the interview data was the need for participants to improve their capacity to use GenAI in nursing research, which also gives free rein to their imagination for the future development and exploration of GenAI. It reflected the willingness of the participants to integrate the wisdom of GenAI into research to promote the advancement of nursing research.

### Reinforcement Needs for GenAI Literacy

There was a significant need for GenAI training in nursing research in China. Participants agreed that nurse researchers need comprehensive training in GenAI, including principles, functions, and interactive skills, which can help them improve efficiency and clarify the correct application concepts. This study defines these capabilities as “GenAI literacy.” They also provided valuable suggestions on the details of the training curriculum.

*I think the training should teach us how to explore the value of GenAI fully. Very often, I felt like I was interacting with a teacher in class because questions could be answered step by step, so it would be useful to learn how to optimise call words to interact with it.*

[S7]

*I prefer to do a case study.…You gave me a statistical method for quantitative research, and I learn how to use GenAI step by step until the article is completed. It is like a workshop with hands-on activities.*

[C6]

#### Development of Nursing Research GenAI

The participants expressed well-informed opinions about the future development of GenAI, including an ambiguous future that considers users’ unique requirements and the significant idea that the value generated by GenAI should benefit clinical practice. As the field of nursing becomes more extensive and deeply intertwined with other disciplines, participants expressed a desire to create a nursing-specific GenAI, developed by a multidisciplinary team, based on the academic achievements of GenAI combined with pragmatic needs to meet the needs of nurse researchers for multiple knowledge and personalized learning.

*I think that the scientific and educational outcomes of GenAI, when put into practice, will bring benefits to patients and society that can be said to be immeasurable, and I am very optimistic about this.*

[F4]

*Is there a corpus for the nursing profession, or some database, that would allow us to search more accurately for future nursing research knowledge for nurses? That way, the information nurses receive will be more reliable.*

[C3]

#### Urgent Need for AI-Generated Content Detection Tools

This subtheme drew nurse researchers’ attention to providing forward-looking insights into the urgent need for GenAI detection tools. They hoped that the use of GenAI would be normatively constrained to some extent by GenAI detection tools, thereby reducing concerns about academic misconduct.

*If a researcher has used GenAI but has not declared it, and therefore does not know about the development of tools to identify GenAI content...Nevertheless, fighting AI with AI is a good idea.*

[S7]

## Discussion

### Summary of Results

The findings of this study genuinely reflect the multidimensional experiences, perceptions, and future development regarding the use of GenAI in nursing research by nurse researchers. In general, nurse researchers and GenAI have a symbiotic relationship. At present, nurse researchers have rich experiences in using GenAI, covering many aspects of nursing research, and their perceptions of GenAI vary. Despite the controversies surrounding GenAI as an emerging tool, including potential ethical risks, benefits, and limitations of its use, nurse researchers still hold a generally positive attitude toward the use of GenAI in nursing research. In the future, GenAI should be developed for nursing research and enhance the GenAI usage capabilities of nurse researchers.

### GenAI Is an Enhancer Not a Replacer

Overall, nurse researchers have shown a high interest and held a positive attitude toward GenAI. On the specific level of user experience, the nurse researchers included in this study widely regarded GenAI as a groundbreaking auxiliary tool for nursing research. This view is based on 2 dimensions of thought: one is “groundbreaking” and the other is “assistive.” Groundbreaking in the sense that applying GenAI to nursing research is an irresistible trend and its emergence has epoch-making significance. Compared to traditional research tools such as search engines and statistical software, GenAI boasts advantages such as simple interaction, convenience and speed, and rapid iteration, which is consistent with the views of existing research [35]. Researchers also acknowledged that GenAI can transcend linguistic barriers and enhance the efficiency of their writing process, which is in line with another research [36]. The “assistive” nature is the prerequisite for the “groundbreaking” existence, meaning that GenAI plays a groundbreaking role in auxiliary scientific research tasks, such as knowledge acquisition, inspiring ideas, and aiding those with insufficient research capabilities as an “ability enhancer,” rather than an “ability replacer.” Through the “enhancement” of nursing research, nurse researchers and GenAI are expected to pioneer a new research path, ultimately reshaping the nursing research paradigm.

### Use Technological Development to Address Technological Deficiencies

While GenAI has shown significant advantages in nursing research, it still faces various limitations at the current level of technological development. Among these, a criticized issue by nurse researchers is the fabrication by GenAI. This issue arises because GenAI fundamentally relies on advanced statistical models to predict responses to user input, without actual thinking [37]. Hence, its responses might not be true in real-world scenarios [38]. On the other hand, it is a challenge for GenAI to shoulder the burden of fully assuring the accuracy in nursing research. The training corpus behind GenAI mainly comes predominantly from the internet; therefore, its accuracy within the field of nursing research cannot be guaranteed. Moreover, there are some additional scruples put forward by participants, such as poor interaction methods and barriers related to the internet and economics, yet it also represented areas where they hoped to develop a GenAI specifically for nursing research. To achieve this goal, the participants of this study believe that nurse researchers need to work closely with the technicians who develop GenAI. To concretize this collaboration, nurse researchers are responsible for the selection and tagging of the nursing professional corpus and for exploring and surveying the needs of stakeholders in the nursing research; based on the construction of the professional corpus and needs exploration, technicians can optimize the performance of nursing research GenAI through techniques such as supervised fine-tuning [39], knowledge graph [40], and retrieval-augmented generation [41], reducing the occurrence of “AI hallucinations,” enhancing its timeliness, interpretability, and developing a multimodal GenAI that meets the diverse needs of nurse researchers [42]. At the same time, nurse researchers should also enhance their critical thinking to further add a humanistic touch to the content of GenAI.

### Joint Regulations and Technology Address Ethical Issues

From an ethical perspective, the writing of academic research reports, which primarily rely on textual and visual presentation, is facing multiple challenges due to the powerful generative capabilities of GenAI [18,43]. On one hand, some nurse researchers who lack self-discipline may use GenAI to generate false research results and reports. Studies have shown that humans are unable to distinguish between research reports written by humans and those generated by GenAI [44], turning inherent advantages of GenAI into hotbeds for academic misconduct. Consequently, these research findings are likely to be translated into evidence and ultimately applied in clinical settings, leading to a series of nursing ethical issues and endangering patient life and health. This concern is consistent with that raised in another study [45]. On the other hand, researchers have different views on whether GenAI should be recognized as an author. Currently, it is widely accepted across most academic structures and publishing organizations that “GenAI cannot be accredited as an author” [20], and some nurse researchers introduced somewhat progressive thoughts, arguing that GenAI should be perceived and endowed with rights akin to human authorship. This could be attributed to a potential unfamiliarity with the technical capabilities and underlying principles of GenAI, and it also reflects nurse researchers’ recognition of GenAI capabilities. However, these controversies should not lead to the prohibition of the legitimate use of GenAI in nursing research. Some research teams have developed tools for detecting AI-generated content [46], which helps to mitigate the ethical issues associated with GenAI. At the same time, international cooperation should also be promoted to develop unified application specifications for GenAI in nursing research, assess potential ethical risks, and provide guidance on risk mitigation. As nurse researchers, they should receive specialized training on the ethical use of GenAI. The training content should cover not only the technical aspects but also the ethical and legal implications. The collaborative development of humans and machines can only solve the ethical dilemmas in human-machine interaction.

### Nurse Researchers’ Anxiety About Being Replaced

Moreover, participants had varied opinions on whether GenAI could replace their jobs in the future. The majority of participants believed that GenAI can merely serve as an auxiliary tool in nursing research; nonetheless, it cannot supersede researchers in terms of creative tasks such as topic selection and making research protocol, a viewpoint that coincides with an existing study [47]. However, some nurse researchers expressed concerns that GenAI might replace their jobs in the future. There are multiple reasons for this phenomenon: media portrayals have exaggerated the capabilities of GenAI, and many nurse researchers have not delved into the principles and limitations of GenAI’s capabilities, mistakenly believing that GenAI has become a thinking-intelligent entity, thus leading to current anxieties. A study shows that this anxiety also exists within the student population [48]. These anxieties all stem from the incomplete understanding of GenAI, which shows the necessity of promoting and popularizing knowledge about GenAI.

### Enhancing the Overall Competence Is Key

Within another understanding of human-machine interplay by nurse researchers, the efficacy of GenAI is intimately tied to the proficiency of the user. First, people who lack scientific literacy and information discernment ability may be misled by GenAI’s answers, which could be consistent with another study [49], contrary to the expectation that it can provide valid information and increase the efficiency of research. Second, nurse researchers’ grasp of GenAI application techniques (prompt engineering) fundamentally influences the quality of studies using GenAI [50]. This enlightens us that the enrichment of the nurse researchers’ basic scientific literacy is key to achieving the full potential of GenAI, which should be complemented by training prompt skills. Another noteworthy point is that different nursing research groups exhibited variations in their perceptions and use of GenAI. The reasons behind these distinctions relate to the participants’ internal characteristics as well as external factors such as their field of study and work pressure. Essentially, these factors reflect the GenAI literacy of nurse researchers. This is consistent with the results of a survey on AI literacy of nursing staff [51]. In the future, GenAI literacy evaluation tools should be developed based on today’s AI literacy evaluation system to lay a foundation for enhancing the ability of nurse researchers to use GenAI in nursing research.

### Artificial General Intelligence for Nursing Research Is the Future Direction

Participants in this study have identified a diverse range of specific application scenarios for GenAI within nursing research, and a survey conducted by *Nature* has also confirmed this phenomenon [52]. This diversity aligns with the future development directions of GenAI, which is toward artificial general intelligence (AGI) [53]. However, GenAI still performs poorly or cannot be applied in some nursing research scenarios at this stage. Therefore, the ideal development for GenAI’s application in nursing research is to achieve an AGI that covers general needs. In the future, the GenAI for nursing research should not be a simple chatbot based on text and video. Instead, it should be an Agent AI with multimodal capabilities, the ability to interact with the real world, reasoning ability, and the capacity to independently complete some scientific research tasks [54]. This kind of nursing research can empower the improvement of the efficiency and quality of nursing research, which will also enhance the quality of nursing, ultimately benefiting patients.

### Strengths and Limitations

This study is relatively innovative in adopting a qualitative research method to explore the different nursing research groups’ experiences of using GenAI in nursing research, which can deeply explore the nurse researchers’ experiences and perceptions of GenAI as an emerging phenomenon. Meanwhile, this study selected 3 groups of participants which cover the common segments of the nursing research community, ensuring the comprehensiveness of the data. The research was limited by some constraints that might influence the outcomes. The study’s participants primarily hail from academic and medical institutions in Beijing and Shanghai, China. On the one hand, the economic affluence of these 2 developed cities may not mirror the realities of other developing regions and countries. On the other hand, there are certain thresholds to using high-performance GenAI, such as ChatGPT in China, leading to a general shortfall in advanced GenAI experiences among the researchers.

### Implications of Research Findings for Policy and Future Research

The findings of this research reflected the current state of GenAI usage among nurse researchers, clarifying their experiences and perceptions with GenAI. These insights are poised to facilitate rational application and positive evolution of GenAI within nursing research, offering guidance for policy making and future research directions. The key points are as follows:

1. Establishing norms for the use of GenAI: norms for the use of GenAI should be swiftly established through international collaboration, ensuring it adheres to academic and nursing ethics while fully leveraging GenAI’s strengths to enhance research efficiency and bridge language gaps, accompanied by the introduction of relevant academic standards and policies.

2. Construct a GenAI literacy cultivation and evaluation system: in the future, systematic GenAI training tailored for nurse researchers with individual characteristics should be conducted, enabling them to use GenAI correctly and effectively in nursing research. At the same time, nurse researchers should also construct evaluation tools for GenAI literacy to assist in the cultivation of GenAI literacy.

3. Interdisciplinary cooperation to develop nursing research AGI: nurse researchers need to collaborate closely with technology professionals. Nurse researchers are responsible for corpus control, user needs assessment, and efficacy validation, while technical staff are tasked with model training to create high-performance AGI tools specifically for nursing research.

### Conclusions

The application of GenAI in nursing research is beginning to emerge, but given the different reasons, this “symbiotic journey” between GenAI and nurse researchers is unlikely to be smooth sailing. In this journey, nurse researchers should continuously learn to enhance their scientific literacy, digital literacy, and prompt skills. They should join hands with academic and publishing institutions to leverage the advantages of GenAI fully. At the same time, nurse researchers should also enhance their critical thinking and awareness of academic and nursing ethics to avoid academic misconduct and clinical problems caused by GenAI. Collaborating with technicians to develop GenAI that meets the diverse needs of the nursing research community is also necessary. Ultimately, nurse researchers will join hands with GenAI, improving the efficiency and quality of nursing research.

## Supplementary material

10.2196/65523 Multimedia Appendix 1 COREQ (Consolidated Criteria for Reporting Qualitative Research) checklist.

## Abbreviations

AGI: artificial general intelligence
AI: artificial intelligence
COREQ: Consolidated Criteria for Reporting Qualitative Research Checklist
GenAI: generative artificial intelligence
