# Supplementary material for: Nurse Researchers’ Experiences and Perceptions of Generative AI: Qualitative Semistructured Interview Study
Source: J Med Internet Res. 2025 Aug 25;27:e65523. doi: 10.2196/65523 (PMC12377238; doi:10.2196/65523)
Supplement: Multimedia Appendix 1 [file jmir-v27-e65523-s001.pdf]

COREQ checklist

Consolidated criteria for reporting qualitative studies (COREQ): 32-item checklist

Developed from:
Tong A, Sainsbury P, Craig J. Consolidated criteria for reporting qualitative research (COREQ): a 32-item checklist for interviews and focus groups. International Journal for Quality in Health Care. 2007. Volume 19, Number 6: pp. 349 – 357

Item No	Guide Questions/Description	Reported on Page #
**Domain 1: Research team and reflexivity**		
**Personal Characteristics**		
1. Interviewer/ facilitator	Which author/s conducted the interview or focus group?	Pg. 7
2. Credentials	What were the researcher's credentials? E.g., PhD, MD	Pg. 7
3. Occupation	What was their occupation at the time of the study?	Pg. 7
4. Gender	Was the researcher male or female?	Pg. 7
5. Experience and training	What experience or training did the researcher have?	Pg. 7
**Relationship with participants**		
6. Relationship established	Was a relationship established prior to study commencement?	Pg. 6
7. Participant knowledge of the interviewer	What did the participants know about the researcher? e.g. personal goals, reasons for doing the research?	Pg. 6
8. Interviewer characteristics	What characteristics were reported about the interviewer/facilitator? e.g. Bias, assumptions, reasons and interests in the research topic	Pg. 6
**Domain 2: study design**		
**Theoretical framework**		
9. Methodological orientation and Theory	What methodological orientation was stated to underpin the study? e.g. grounded theory, discourse analysis, ethnography, phenomenology, content analysis	Pg. 6, Pg. 8
**Participant selection**		

Item No	Guide Questions/Description	Reported on Page #
10. Sampling	How were participants selected? e.g., purposive, convenience, consecutive, snowball	Pg. 6
11. Method of approach	How were participants approached? e.g., face-to-face, telephone, mail, email	Pg. 6
12. Sample size	How many participants were in the study?	Pg. 9
13. Non-participation Setting	How many people refused to participate or dropped out? Reasons?	N/A
14. Setting of data collection	Where was the data collected? e.g., home, clinic, workplace	Pg. 6
15. Presence of nonparticipants	Was anyone else present besides the participants and researchers?	N/A
16. Description of sample	What are the important characteristics of the sample? e.g. demographic data, date	Pg. 9-11
**Data collection**		
17. Interview guide	Were questions, prompts, and guides provided by the authors? Was it pilot tested?	Pg. 7
18. Repeat interviews	Were repeat interviews carried out? If yes, how many?	N/A
19. Audio/visual recording	Did the research use audio or visual recording to collect the data?	Pg. 7
20. Field notes	Were field notes made during and/or after the interview or focus group?	Pg. 7
21. Duration	What was the duration of the interviews or focus group?	Pg. 9
22. Data saturation	Was data saturation discussed?	Pg. 7
23. Transcripts returned	Were transcripts returned to participants for comment and/or correction?	N/A
**Domain 3: analysis and findings**		
**Data analysis**		
24. Number of data coders	How many data coders coded the data?	Pg. 8
25. Description of the coding tree	Did the authors provide a description of the coding tree?	N/A
26. Derivation of themes	Were themes identified in advance or derived from the data?	Pg. 8
27. Software	What software, if applicable, was used to manage the data?	Pg. 8

Item No	Guide Questions/Description	Reported on Page #
28. Participant checking	Did participants provide feedback on the findings?	Pg. 9
**Reporting**		
29. Quotations presented	Were participant quotations presented to illustrate the themes/findings? Was each quotation identified? e.g., participant number	Pg. 11-21
30. Data and findings consistent	Was there consistency between the data presented and the findings?	Pg. 11-21
31. Clarity of major themes	Were major themes clearly presented in the findings?	Pg. 11-21
32. Clarity of minor themes	Is there a description of diverse cases or a discussion of minor themes?	Pg. 11-21
